# In Situ Investigation of the Motion Behavior of Graphene on Liquid Copper

**DOI:** 10.1002/advs.202100334

**Published:** 2021-07-08

**Authors:** Luyang Wang, Yu Ding, Xiaozheng Wang, Runze Lai, Mengqi Zeng, Lei Fu

**Affiliations:** ^1^ College of Chemistry and Molecular Sciences Wuhan University Wuhan 430072 China

**Keywords:** graphene, in situ investigation, liquid metal, motion behavior, self‐assembly

## Abstract

The in situ investigation of the dynamic growth process and novel assembly phenomena of graphene on liquid copper (Cu) is of great significance to deeply understand the special behavior of graphene and self‐assembly mechanism. Here, the direct observation of the graphene growth and motion behavior on liquid Cu via in situ imaging is reported. Evidence of graphene movement on liquid Cu is offered and it is demonstrated that the translation and rotation behaviors of graphene are affected by the surface condition of liquid Cu. The self‐assembly process of graphene array is also revealed by capturing the dynamic changes of graphene in real‐time. Further analysis highlights the importance of surface energy of liquid Cu and the interaction between graphene building blocks during the self‐assembling process. The growth parameters are also investigated to flexibly control the assembly configuration of graphene arrays. This work provides an insight into the mechanism of graphene motion and assembly behavior that can be used to guide the controllable manipulation of 2D materials and on‐demand fabrication assembly structures with desired properties.

## Graphical abstract

This work develops an in situ investigation system to study the growth and self‐assembly of graphene on liquid copper in real‐time. The translation and rotation behavior of graphene are captured for the first time, which is attributed to the fluidity of liquid copper surface. The self‐assembly mechanism is further revealed, enabling the precise synthesis of various assembly structures.

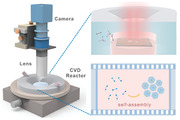

## 1 Introduction

The controllable synthesis of large‐size, defect‐free graphene is a main challenge toward its practical application.^[^ ^1^ ^]^ Chemical vapor deposition (CVD) has emerged as a promising approach for synthesizing high‐quality graphene based on metal catalysts.^[^ ^2^, ^3^ ^]^ Currently, liquid metal catalysts (e.g., molten Cu) with a quasi‐atomically flat surface, show great potential in synthesizing uniform graphene with significantly higher quality and faster growth speed.^[^ ^4^, ^5^, ^6^, ^7^, ^8^, ^9^, ^10^, ^11^, ^12^ ^]^ Meanwhile, the intrinsic fluidity of liquid metal provides a platform for precise manipulation of graphene to achieve complex assembly behaviors.

In our previous works, we have achieved the controllable synthesis of a series of 2D atomic crystal arrays based on liquid metals.^[^ ^13^, ^14^, ^15^, ^16^ ^]^ We discovered that graphene could assemble spontaneously into a super‐ordered structure on liquid Cu surface.^[^ ^13^ ^]^ Research on this self‐assembly behavior of graphene is of great significance to scalable and cost‐effective fabrication of integrated devices. However, the current exploration of the growth and self‐assembly mechanism only relies on ex situ experiments and theoretical calculations.^[^ ^13^, ^17^ ^]^ The experimental setups of CVD are similar to “black box” in most cases. There are some scientific issues still unclear, such as the dynamic growth and self‐assembly process, and the thermodynamics and kinetics mechanism of self‐assembly behavior on liquid Cu. In situ research enables direct visualization of the dynamic process during the crystal growth and evolution,^[^ ^18^, ^19^, ^20^, ^21^, ^22^, ^23^ ^]^ which is expected to help understand the special behavior of graphene on liquid Cu and provide guidance for precisely synthesizing various assembly structures.

Here, we investigate the growth and motion behavior of graphene on liquid Cu via in situ imaging for the first time. We offer explicit evidence to the movability of graphene on liquid Cu at high temperature. The translational and rotational motion of graphene are captured during the growth and assembly, which are related to the condition of liquid Cu surface. We show the dynamic process of graphene self‐assembly behavior and further reveal the self‐assembly mechanism. We demonstrate that both the minimum surface energy and interaction between graphene are crucial to the formation of aligned graphene arrays via self‐assembling. We further explore the influence of the growth parameters on the growth and self‐assembly of graphene. Our work highlights the unique potential of an in situ technique that enables improved understanding of mechanisms in the CVD process. We believe that our in situ investigation could guide the precise assembly of 2D atomic crystals and promote applications in integrated systems.

## 2 Results and Discussion

**Figure 1:**
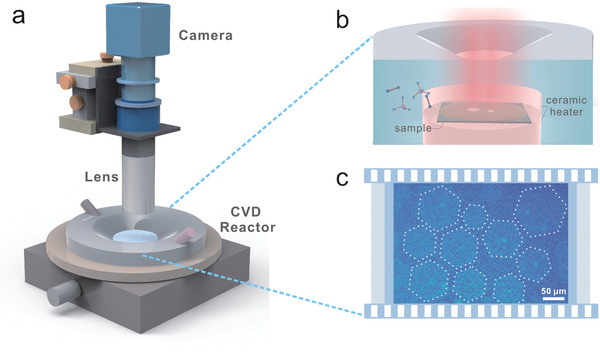
In situ growth of graphene on liquid Cu. a) Schematic illustration of the in situ investigation system for imaging graphene growth behavior. b) Schematic of the growth of graphene single crystal in CVD reactor. c) Rad‐OM image of as‐grown graphene on liquid Cu obtained at 1100 °C.

For studying growth and motion behavior of graphene on liquid Cu, we built an in situ investigation system (**Figure** **1**a and Figure S1, Supporting Information). A custom‐made CVD reactor with a transparent window was fabricated for growing graphene and providing a platform for direct observation. Radiation‐mode optical microscopy (Rad‐OM) enables real‐time imaging of the graphene behavior.^[^ ^20^ ^]^ The schematic of in situ growth of graphene in a CVD reactor is shown in Figure 1b. In the reaction chamber, Cu foil located on tungsten (W) foil was placed inside the cylindrical groove in the center of the ceramic body, which was directly below the observation window. Thermal energy is provided by Joule heating from the winding electric heating wire which is wrapped in the underneath and sides of ceramic groove. With a temperature range from ambient to 1200 °C, the chamber is ideally suited to the study of liquid Cu system. The dynamic melting process of Cu was recorded in Figure S2 and Video S1 (Supporting Information). The Cu foil melted at high temperature (≈1083 °C) and was firmly anchored on the W foil. After the phase transition to the liquid state, a smooth Cu surface without grain boundary was obtained. Then, graphene was grown on liquid Cu by introducing CH_4_. Based on different emissivity between graphene and Cu, they could be distinguished at high temperature.^[^ ^20^, ^24^, ^25^, ^26^ ^]^ The typical Rad‐OM image of as‐grown graphene is shown in Figure 1c. Graphene single crystals with hexagonal shape were visible, which was conducive to subsequent observation. Meanwhile, more ex situ characterizations were presented to verify the high quality of as‐grown graphene (Figure S3, Supporting Information).

**Figure 2:**
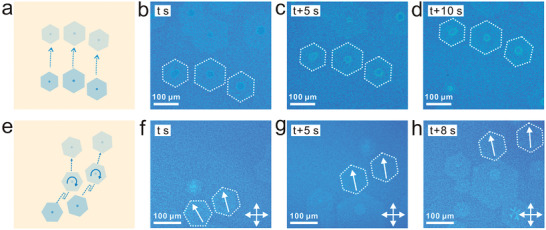
Real‐time observing motion behavior of graphene flakes on liquid Cu surface. a) Schematic of graphene flakes translational motion on liquid Cu. b–d) In situ Rad‐OM images of graphene during the translational movement. All the images were picked up from Video S2 (Supporting Information). e) Schematic of graphene changing the direction movement. f–h) In situ Rad‐OM images of graphene during the rotational movement. All the images were picked up from Video S3 (Supporting Information).

The as‐fabricated in situ system opens a new path to explore the dynamic information of graphene on liquid Cu. Based on this, we visualized the motion behavior of graphene on liquid Cu in real‐time. Compared with solid Cu, liquid Cu with the smooth surface and high fluidity enables the motion behavior of graphene. Meanwhile, the growth and motion of graphene on liquid Cu is more flexible and controllable without the disturbances of grain boundaries, steps and different crystal faces in solid Cu.^[^ ^27^, ^28^, ^29^, ^30^ ^]^ In our experiments, we found that the motion behavior of graphene was closely related to the surface condition of liquid Cu.^[^ ^31^, ^32^ ^]^ As shown in **Figure** **2**a–d and Video S2 (Supporting Information), several graphene flakes moved almost parallel on the surface of liquid Cu. We think the motion behavior is triggered by the surface flow of liquid Cu. The dotted line with arrow in Figure 2a shows the translation direction and motion path of graphene. The positions of three graphene flakes with 5 s intervals are shown in Figure 2b–d, and the distances (which refer to the distance between the centers of two graphene) between three graphene flakes were measured. The distances were almost constant during the translational movement (Figure S4, Supporting Information), indicating their approximate parallel motion as a whole. The motion direction of graphene could be changed when the liquid Cu surface was slightly disturbed (Figure 2e–h and Video S3, Supporting Information). Meanwhile, graphene rotated on the surface of liquid Cu as the motion direction changed. The schematic diagram of graphene changing the direction movement is shown in Figure 2e and the dashed line with arrows represents the motion path of graphene. The curves with arrows on graphene represent the direction of graphene rotation. In this process, the graphene first translated in the upper right direction (Figure 2f). Under a slight disturbance on liquid Cu, graphene flakes moved backward briefly and then continued to move forward in the initial movement direction. Subsequently, they changed the direction and moved upward. The white arrow on graphene is used to indicate the rotation angle of graphene. The graphene on the left rotated 18° clockwise, and the right graphene rotated 6° clockwise (Figure 2g). In the following movement, the right graphene continued rotating 5° clockwise (Figure 2h). The translation and rotation of graphene on liquid Cu suggest the flexibility of motion behavior, which is conducive to timely adjustment of the movement path by regulating the surface condition of liquid Cu.

**Figure 3:**
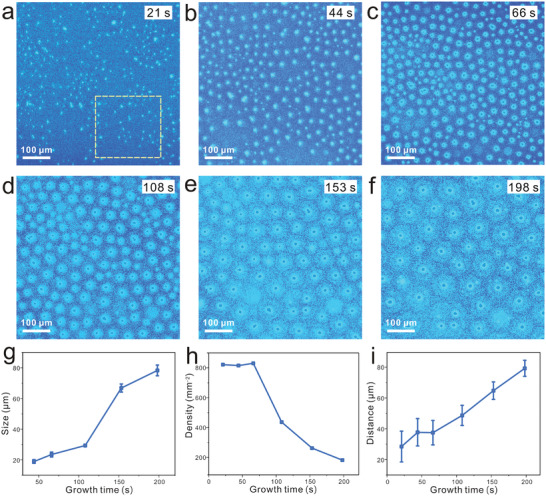
Real‐time observing the self‐assembly of graphene on liquid Cu. a–f) In situ Rad‐OM images of the growth and self‐assembly process. All the images were the partial area picked up from Video S4 (Supporting Information). g) Plot of the size of graphene as a function of growth time. The analyzed region is the marked area in (a). The error bars represent the standard error from measurements of 25 graphene. h) Plot of the density of graphene as a function of growth time. The analyzed region is the marked area in (a). i) Plot of the distance between adjacent graphene as a function of growth time. The analyzed region is the marked area in (a). The error bars represent the standard error from measurements of 45 graphene.

In addition to the translation and rotation behaviors of graphene, complex behavior can also be realized based on liquid Cu. We achieved the in situ observation of graphene assembly behavior on liquid Cu. The information of nucleation, growth, and assembly process can be extracted directly from the in situ recorded videos captured by Rad‐OM (Video S4, Supporting Information). Here, the growth time is defined as the duration time from the moment when the nuclei is visible to the time when the graphene array is well‐assembled. As shown in **Figure** **3**a, the nucleation of graphene was clearly observed at 21 s, and the nuclei occurred simultaneously, which were in random distribution. At the initial growth stage, the outline of graphene gradually appeared with the seed as the center. Meanwhile, graphene moved to achieve uniform distribution (Figure 3b). Graphene further grew to ≈30 µm and began to show a trend of regular arrangement on account of their own translation and rotation behaviors (Figure 3c). As the growth time increased, graphene continually grew and was slightly shifted and rotated to obtain a more ordered arrangement (Figure 3d). The array trend of graphene was gradually obvious. The self‐aligned graphene array was formed (Figure 3e) and the size of graphene continued increasing to about 80 µm (Figure 3f). To better understand the assembly process, we chose a typical region as indicated by the white dotted square in Figure 3a to analyze the evolution of the graphene array. The density, size, and distance of graphene single crystals as a function of growth time are shown in Figure 3g–i. As the growth time increased in the initial stage, the size of graphene increased (Figure 3g), and the density which represents the number of graphene in unit area changed slightly (Figure 3h), demonstrating that the arrangement of graphene single crystals dominated in the early stage. Subsequently, as graphene grew rapidly, the density decreased, corresponding to Figure 3d–f, which was attributed to out‐of‐view expansion of the growing graphene. We further proved that the arranged graphene array remained almost unchanged as it expanded toward the edge of liquid Cu (Figure S5 and Video S5, Supporting Information). The distance between adjacent graphene also reflected the arrangement trend of graphene as shown in Figure 3i. Owing to the uneven distribution of graphene at the initial stage, the graphene flakes tended to move and arrange. This process involved the dispersion of initially aggregated graphene single crystals, leading to the increase in distance from 21 to 42 s. In order to reach a uniformly arranged configuration, graphene would gather from the surrounding to supplement the selected area so that the density increased and distance between graphene decreased. As the size of graphene increased, the distance increased in the later growth. The decreased standard errors of the distance between adjacent graphene demonstrated the order degree of graphene increased during the growth and assembly process. Moreover, the merged process of graphene arrays by increasing the growth time was investigated, which is shown in Figure S6 in the Supporting Information.

**Figure 4:**
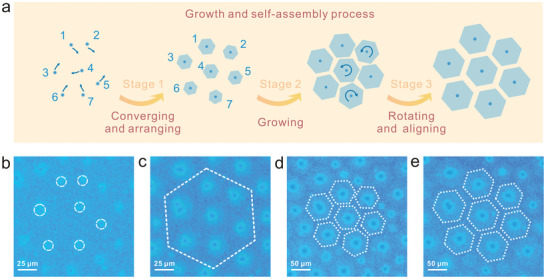
Self‐assembly mechanism of aligned graphene arrays. a) Schematic of growth and self‐assembly process on liquid Cu. b–e) Rad‐OM images of graphene array evolution at 24, 52, 141, 181 s, respectively. All the images were picked up from Video S4 (Supporting Information).

Our results directly presented the dynamic process of growth and self‐assembly of graphene. In order to get an improved understanding of self‐assembly mechanism, the detailed motion behavior between the building blocks was further explored. We focused on seven adjacent building blocks from Video S4 (Supporting Information). The schematic of the formation mechanism of aligned graphene arrays is illustrated in **Figure** **4**a, the directions of these motions for graphene 1–7 are indicated by curved arrows. Figure 4b–e indicates representative moments in the formation of the array. At stage 1, the building blocks with small size were in random distribution (Figure 4b), they would spontaneously converge and arrange on the liquid surface to reach a well‐arranged configuration, which was attributed to the minimum surface energy principle.^[^ ^33^, ^34^, ^35^ ^]^ The building blocks within the dotted hexagonal line indicated the uniform distribution (Figure 4c). Then, the as‐arranged graphene flakes continued to grow at stage 2, the gap between the adjacent graphene decreased with the size increasing, and the relative position between graphene slightly changed owing to the interaction between adjacent graphene (Figure 4d). During the self‐assembly process, we discovered that there was a gap between adjacent graphene flakes so that they would not merge together. As the gap between graphene decreased, the repulsive electrostatic interaction between graphene flakes became more obvious at stage 3,^[^ ^13^, ^17^ ^]^ the graphene would rotate to reach the aligned configuration and translate to keep the balance of arrays, corresponding to Figure 4e.

In situ system with real‐time feedback facilitates the exploration of nucleation and growth under different growth parameters, which can guide the control of the assembly configurations of graphene arrays. We studied the influence of CH_4_ flow rate in the in situ system. The nucleation density and rate as the function of the CH_4_ flow rate were explored in Figure S7, Supporting Information. With the CH_4_ flow rate increasing from 0.6 to 2 sccm, the nucleation time decreased and nucleation density increased, which is attributed to the formation of more activated carbon species.^[^ ^36^ ^]^ Moreover, the CH_4_ flow rate can affect the size and arrangement of graphene. As shown in Figure S8 in the Supporting Information, excessively low CH_4_ flow would make the precursor supply insufficient, which could cause the small size and uneven distribution of as‐grown graphene (Figure S8a, Supporting Information). Proper CH_4_ flow rate could bring suitable nucleation density and uniform size, resulting in aligned graphene arrays (Figure 3 and Figure S8b, Supporting Information). The graphene tended to grow into mutilayers under high CH_4_ flow (Figure S8c, Supporting Information). Besides, the distance between the adjacent building blocks and the size of graphene arrays can be controlled by flexibly adjusting the growth time. As shown in Figure S9 in the Supporting Information, as the growth time was increased from 2 to 3 min, the distance was about 44.7, 24.1, 15.2 µm, respectively. The graphene size increased with the growth time increasing, the mean size of graphene single crystals was about 67.9, 81.7, and 93.6 µm, respectively.

## 3 Conclusion

In summary, we demonstrate the motion behavior and the assembly process of graphene on the liquid Cu via in situ imaging for the first time. The high fluidity of liquid Cu enables graphene to translate and rotate on liquid surface. The real‐time observation of dynamic evolution of graphene arrays further confirms the self‐assembly mechanism. Owing to the minimum surface energy principle, graphene tends to be evenly distributed on the surface of liquid Cu to maintain the most stable state. The interaction between graphene units facilitates the accurate aligned configuration. Besides, the configuration of graphene arrays can be flexibly regulated by controlling the growth parameters. Our findings give more inspirations to on‐demand customize the single crystal assembly via the bottom‐up method to meet more technical requirements.

## 4 Experimental Section

### In Situ Investigation System

The investigation system was composed of two parts, a custom‐made CVD reactor and a radiation‐mode optical microscopy. The custom‐made CVD reactor was consisted of a ceramic heater, a thermal insulator, a water‐cooler, and the gas circuit system. A 1.5 mm thick glass window was located above the heating center for optical observations. The maximum working temperature could reach 1200 °C, with an uncertainty of ±1 °C, which satisfied the graphene growth on liquid Cu. The Rad‐OM was used for in situ optical observations, which was composed of a camera (CS895MU, Thorlabs) and an objective (MY20X‐804, Mitutoyo). The quantum efficiency of camera was 72% over 525 to 580 nm and decreased rapidly to less than 5% at 1000 nm (on the website of the company). A 20× long working distance microscopy objective was applied to focus the samples and prevent the thermal radiation damage due to the high temperature.

### In Situ Melting of Cu

The melting process of Cu under high temperature was investigated using the in situ system. After electrochemically polished, the 0.05 mm Cu foil (99.8% purity, Alfa Aesar) was put on the W (99.5% purity, Alfa Aesar) substrate, which was located in the center of the heating zone. The Cu foil was heated to the melting point and the maximum of the heating rate was less than 30 °C min^−1^. In order to prevent the oxidation of Cu, 200 sccm Ar and 10–30 sccm H_2_ were introduced into the custom‐made CVD reactor during the whole heating process.

### In Situ Growth of Graphene on Liquid Cu Surface

First, the Cu foil was put on the W substrate, which was located in the center of the heating zone. After that, the Cu foil was heated to 1100 °C and the maximum of the heating rate was less than 30 °C min^−1^. When the temperature reached 1100 °C, 1–2 sccm CH_4_ was introduced into the custom‐made CVD reactor for growing graphene. The oxide nanoparticles introduced from the ceramic heater tended to be the nucleation sites, serving as good tracers. After 2–5 min, the CH_4_ was turned off, and the temperature was decreased to room temperature. The whole process was conducted under 200 sccm Ar and 12 sccm H_2_.

### Characterization

Optical images and videos were taken with a radiation‐mode optical microscopy. Raman spectroscopy was performed with WITec alpha 300R system (488 nm excitation wavelength). The low‐resolution transmission electron microscopy (TEM) images were obtained by a high‐resolution TEM system (JEM‐2100) and the operating voltage was 200 kV. Atomic‐resolution scanning transmission electron microscopy (STEM) image was obtained with JEOL ARM‐200F operated at 80 kV.

### Image Processing

All the images were conducted with image manipulation software developed by the authors. The image was subtracted after dulling edges and denoising from all the images for background deduction and the minimum value was added at each pixel to avoid the negative value. Then, all the pixels were stretched equally to form the processed image. The contrast and brightness of images were adjusted in image manipulation software. The adjusted gray‐scale images were converted into RGB images. The final images were achieved via RGB color analysis to distinguish the nuclei, graphene, and liquid Cu more clearly.

## Conflict of Interest

The authors declare no conflict of interest.

## Supporting information

Supporting Information

Supplemental Video 1

Supplemental Video 2

Supplemental Video 3

Supplemental Video 4

Supplemental Video 5

## Data Availability

Research data are not shared.
