# Supplementary material for: In Situ Investigation of the Motion Behavior of Graphene on Liquid Copper
Source: Adv Sci (Weinh). 2021 Jul 8;8(17):2100334. doi: 10.1002/advs.202100334 (PMC8425870; doi:10.1002/advs.202100334)
Supplement: Supplementary file 1 — Supporting Information [file ADVS-8-2100334-s003.pdf]

WILEY-VCH

© 2021 Wiley-VCH GmbH

ADVANCED SCIENCE
Open Access

Supporting Information

for *Adv. Sci.,* DOI: 10.1002/advs.202100334

In-Situ Investigating the Motion Behavior of Graphene on Liquid Copper

Luyang Wang, Yu Ding, Xiaozheng Wang, Runze Lai, Mengqi Zeng, and Lei Fu**

Supporting Information

In-Situ Investigating the Motion Behavior of Graphene on Liquid Copper

Luyang Wang#, Yu Ding#, Xiaozheng Wang, Runze Lai, Mengqi Zeng, and Lei Fu**

#These authors contributed equally to this work.

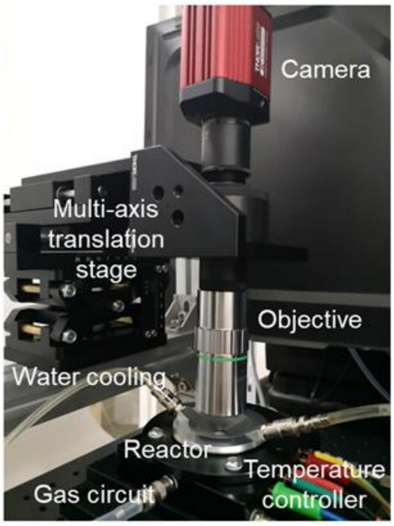

Figure S1. A picture of the custom-made CVD reactor and in-situ observation system. The essential components are labelled on the picture. The focusing of the graphene is performed via moving the nosepiece of the multi-axis translation stage. For movement in X–Y direction, it is achieved by adjusting the rotary knob on the translation stage. In order to maintain stability, the CVD reactor remains immobile during the entire reaction.

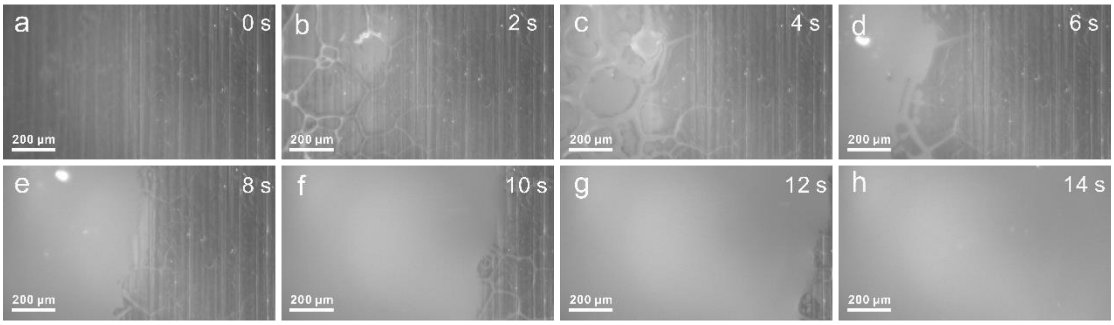

Figure S2. In-situ observing the melting process of Cu. a–h) Rad-OM images of Cu foil during the melting process near melting point at the moments with 2 s intervals. The electro-polishing process flattens the rough surface topography produced by rolling, but cannot completely eliminate rolling marks. When the temperature rose close to the melting point of Cu, Cu foil began to melt from the grain boundary.[1] With the temperature further increasing, the rolling marks gradually disappeared and the surface became smooth rapidly at micrometer scale.

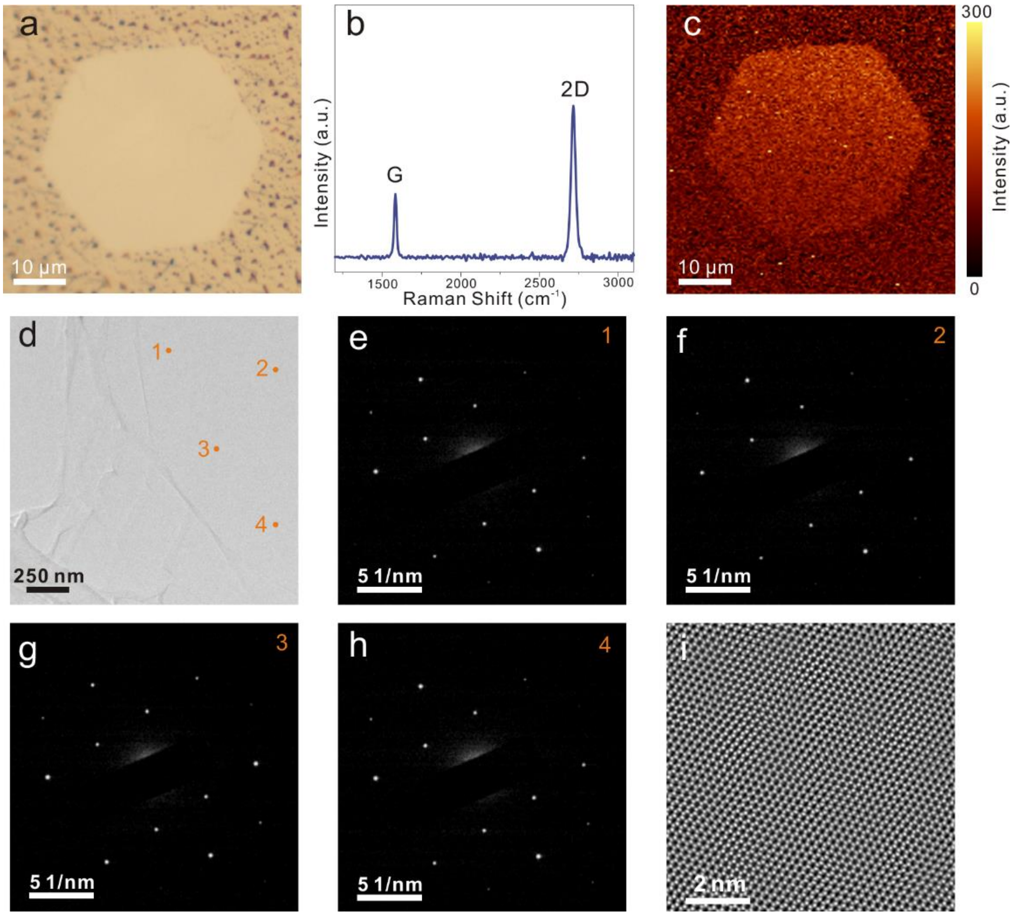

Figure S3. Ex-situ characterizations of as-grown graphene. a) OM image of graphene grown on liquid Cu. b) Raman spectrum of as-grown graphene single crystal in (a). c) Raman mapping of the intensity of the G peak for graphene. The typical Raman spectra indicates that the intensity ratio I_{2D}/I_G is larger than 2, confirming the formation of single-layer graphene. Moreover, the absence of the defect peak also proves the high quality of graphene. The Raman intensity mapping of G peak confirms the uniformity of graphene single crystal. d) Low-resolution transmission electron microscopy (TEM) image of graphene transferred onto a Cu grid. e–h) The selected area electron diffraction (SAED) patterns derived from four different regions marked in Figure S3d. i) Atomic-resolution scanning transmission electron microscopy (STEM) image of graphene.

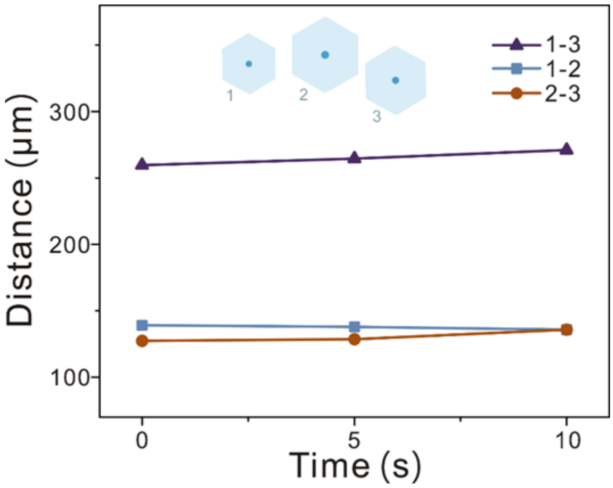

Figure S4. Time evolution of the distance between the graphenes during the translation motion.

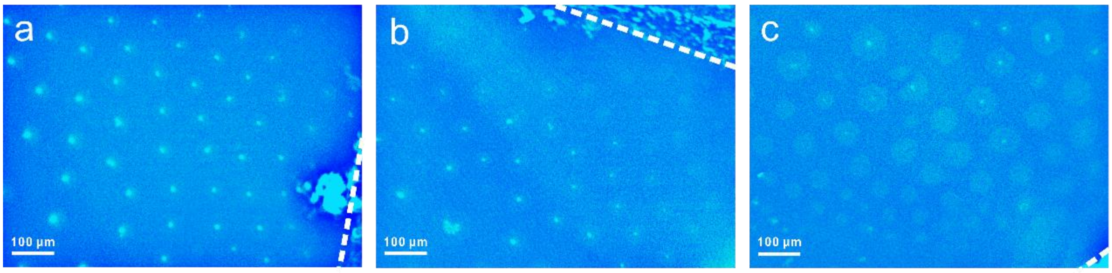

Figure S5. Rad-OM image of the graphene array on the edge of Cu during the self-assembly process. a–c) the graphene arrays were randomly selected from entire liquid Cu edge.

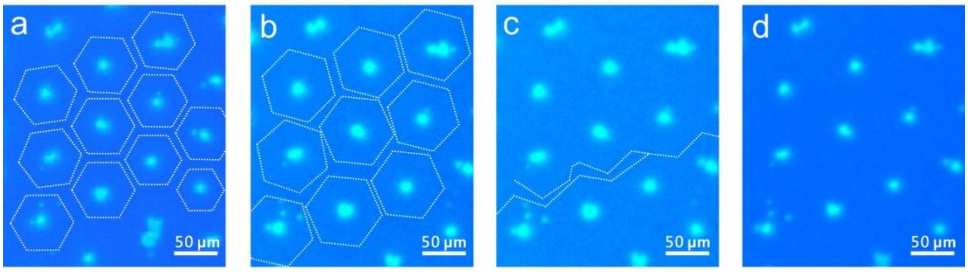

Figure S6. In-situ Rad-OM images of the merged process during graphene growth. The growth moment of Figure R4a is defined as t s, and b–d are t+19, t+29, t+37 s, respectively. The flow of Ar was 200 sccm and the flow of H_2 was 12 sccm. The flow of CH_4 was 1 sccm and the growth time was 4 min.

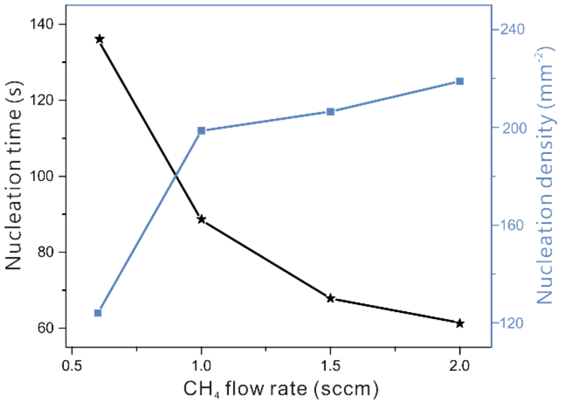

Figure S7. Plot of the nucleation density and nucleation time of graphene on liquid Cu as a function of the CH_4 flow rate. The CH_4 flow rate was 0.6, 1, 1.5, and 2 sccm, espectively. The flow rate of Ar was 200 sccm and the flow of H_2 was 12 sccm.

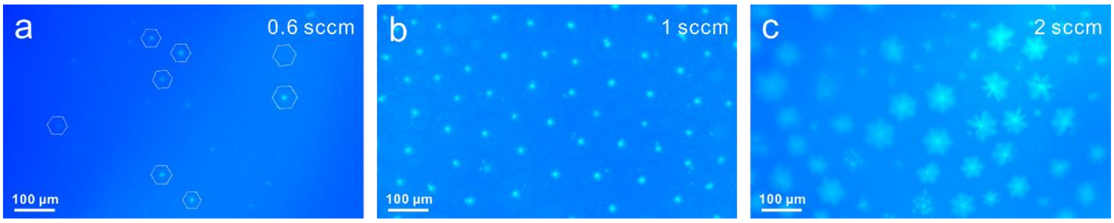

Figure S8. In-situ Rad-OM images of graphene under different CH_4 flow rate. a) 0.6 sccm. b) 1 sccm. c) 2 sccm. In addition, the other growth parameters remained the same. The flow rate of Ar was 200 sccm and the flow rate of H_2 was 12 sccm. The subsequent growth was sustained for 2min 40 s.

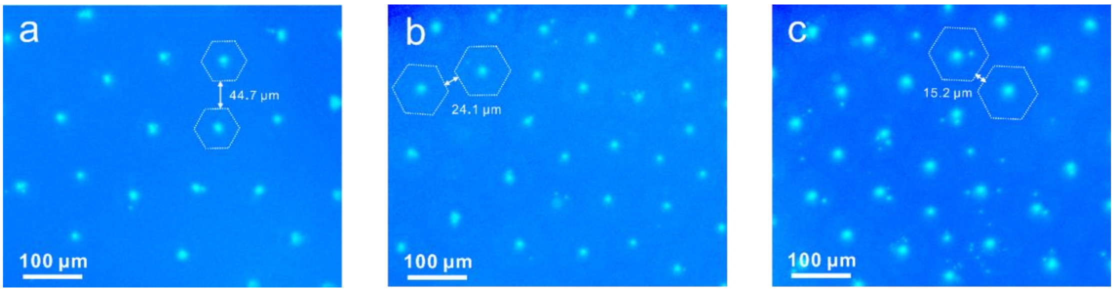

Figure S9. In-situ Rad-OM images of graphene arrays under different growth time. a) 2min. b) 2 min 40 s. c) 3 min. In addition, the other growth parameters remained the same. The flow rate of Ar was 200 sccm and the flow rate of H_2 was 12 sccm. The subsequent growth was sustained under 1 sccm CH_4.

Video information

Video S1. In-situ observing the melting process of Cu in a flow of 200 sccm Ar and 12 sccm H_2 in the process of heating up to 1100 °C. Scale bar, 100 μm. The video plays at normal speed.

Video S2. Real-time observation of the translational movement of graphene on liquid Cu in a flow of 200 sccm Ar and 12 sccm H_2 and a substrate temperature of 1100 °C. 1 sccm CH_4 was introduced into the reactor for 180 s. Scale bar, 50 μm. The video plays at normal speed.

Video S3. Real-time observation of the movement of graphene as the surface of liquid Cu changes in a flow of 200 sccm Ar and 12 sccm H_2 and a substrate temperature of 1100 °C. 1 sccm CH_4 was introduced into the reactor for 180 s. Scale bar, 100 μm. The video plays at normal speed.

Video S4. Real-time observing the self-assembly process of graphene on liquid Cu in a flow of 200 sccm Ar and 12 sccm H_2 and a substrate temperature of 1100 °C. 1 sccm CH_4 was introduced into the reactor for 160 s. Scale bar, 100 μm. The video plays at 5× normal speed.

Video S5. Real-time observing the translational movement of graphene array on liquid Cu in a flow of 200 sccm Ar and 12 sccm H_2 and a substrate temperature of 1100 °C. Scale bar, 100 μm. 1 sccm CH_4 was introduced into the reactor for 120 s. The video plays at 5× normal speed.

References

[1] T. G. Nieh, J. Wadsworth, *JOM* **1992**, *44*, 46.
